# Supplementary material for: Identification of photoperiod-induced specific miRNAs in the adrenal glands of Sunite sheep (Ovis aries)
Source: Front Vet Sci. 2022 Jul 22;9:888207. doi: 10.3389/fvets.2022.888207 (PMC9354845; doi:10.3389/fvets.2022.888207)
Supplement: Supplementary file 1 [file Data_Sheet_1.ZIP › Supplementary file/miRDeep2 file.zip!/miRDeep2 file/Know_Structure/SPLP42A1/oar-mir-1197.pdf]

miRBase precursor : oar-mir-1197
Total read count : 67
oar-miR-1197-5p read count : 1
oar-miR-1197-3p read count : 54
remaining reads : 12

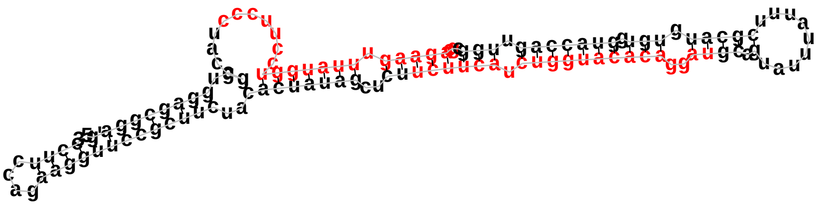

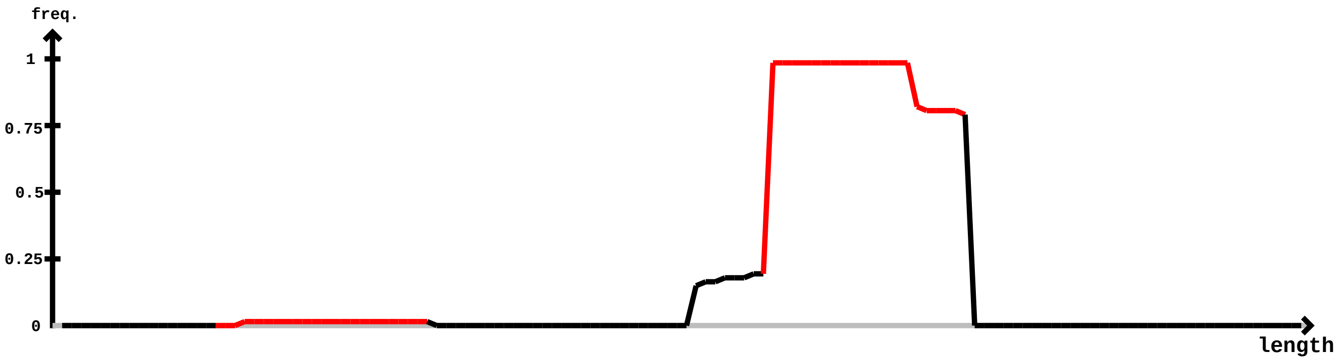


```
                                                                       oar-miR-1197-3p
                oar-miR-1197-5p
5'- gaggcgaggucggcaucccuuccugguauuugaagacgcgguugaccaugguguguacgcuuuauuuaugacguaggacacauggucuacuucuucucgauaucacaucuucgccuuggaagaccuucc   -3'   exp
    ((((((((..(...........(((((((.(((((...(((.(((((((.(((.(((((.........).))))..)))))))))).))))))))..))))))))..)))))))))((((....))))   reads   mm
    ..................cuuccugguauuugaagacg...........................................................................................   1      0
    .................................................................uuaugacguaggacacauggucu.........................................   9      0
    .................................................................uuaugacguaggacacauggucua........................................   1      0
    ..................................................................uaugacguaggacacauggucu.........................................   1      0
    ....................................................................ugacguaggacacauggucu.........................................   1      0
    .......................................................................cguaggacacauggucuacuucu...................................   1      0
    .........................................................................uaggacacauggucuacuuc....................................   1      0
    .........................................................................uaggacacauggucuacuucu...................................   52     0
```
